# Multimorbidity of four cardiometabolic and chronic pulmonary disease groups: prevalence and attributable fraction in US adults, 2007–2012

**DOI:** 10.15256/joc.2017.7.89

**Published:** 2017-03-13

**Authors:** Lisa R. Staimez, Melissa Y. Wei, Min Kim, K. M. Venkat Narayan, Sharon H. Saydah

**Affiliations:** ^1^Hubert Department of Global Health, Rollins School of Public Health, Emory University, Atlanta, GA, USA; ^2^Division of General Medicine and Institute for Healthcare Policy and Innovation, University of Michigan, Ann Arbor, MI, USA; ^3^Department of Epidemiology, Rollins School of Public Health, Emory University, Atlanta, GA, USA; ^4^Department of Medicine, School of Medicine, Emory University, Atlanta, GA, USA; ^5^Centers for Disease Control and Prevention, Division of Diabetes Translation, Hyattsville, MD, USA; ^*^Equal contribution.

**Keywords:** multimorbidity, comorbidity, chronic disease prevalence, population attributable fraction, cardiovas cular disease, diabetes mellitus, chronic kidney disease, chronic obstructive pulmonary disease, hypertension, obesity

## Abstract

**Background:**

Cardiometabolic and chronic pulmonary diseases may be associated with modifiable risk factors that can be targeted to prevent multimorbidity.

**Objectives:**

(i) Estimate the prevalence of multimorbidity across four cardiometabolic and chronic pulmonary disease groups; (ii) compare the prevalence of multimorbidity to that of one disease and no disease; and (iii) quantify population attributable fractions (PAFs) for modifiable risk factors of multimorbidity.

**Design:**

Data from adults aged 18–79 years who participated in the US National Health and Nutrition Examination Survey 2007–2012 were examined. Multimorbidity was defined as ≥2 co-occurring diseases across four common cardiometabolic and chronic pulmonary disease groups. Multivariate-adjusted PAFs for poverty, obesity, smoking, hypertension, and low high-density lipoprotein (HDL) cholesterol were estimated.

**Results:**

Among 16,676 adults, the age-standardized prevalence of multimorbidity was 9.3%. The occurrence of multimorbidity was greater with age, from 1.5% to 5.9%, 15.0% and 34.8% for adults aged 18–39, 40–54, 55–64 and 65–79 years, respectively. Multimorbidity was greatest among the poorest versus non-poorest adults and among blacks versus other races/ethnicities. Multimorbidity was also greater in adults with obesity, hypertension, and low HDL cholesterol. Risk factors with greatest PAFs were hypertension (38.8%; 95% confidence interval [CI] 29.4–47.4) and obesity (19.3%; 95% CI 10.2–28.2).

**Conclusions:**

In the USA, 9.3% of adults have multimorbidity across four chronic disease groups, with a disproportionate burden among older, black, and poor adults. Our results suggest that targeting two *intermediate* modifiable risk factors, hypertension and obesity, might help to reduce the prevalence of multimorbidity in US adults.

## Introduction

Multimorbidity, commonly referred to as the co-existence of two or more chronic diseases in an individual [1–3], is associated with poor health-related quality of life, and increased healthcare utilization and cost, hospital length of stay and readmission, and mortality [4, 5]. Chronic non-communicable diseases, particularly cardiometabolic [6] and chronic pulmonary diseases, are common and fatal diseases in the USA [7]. In 2014, some of the top leading causes of death in the USA included diseases of the heart and cerebrovascular diseases, lower respiratory diseases, diabetes, and kidney diseases, including nephritis, nephrotic syndrome, and nephrosis [8]. Despite the grave consequences of these common diseases, the prevalence and quantification of risk factors using population attributable fractions (PAFs) for multimorbidity in a US-representative general adult population are not well understood. 

Multimorbidity patterns, including a clustering of cardiovascular, metabolic, and psychiatric diseases, share disease pathways that have been described previously [9–11]. However, not all co-occurring diseases may be attributed to common pathophysiologic processes or the natural progression of disease. Unrelated disease patterns have been described as discordant, in which morbidities have largely unrelated pathophysiology and treatment approaches [12]. In contrast, morbidities may also have a shared pathophysiology. Diseases with a shared pathophysiology may also have related or overlapping modifiable risk factors (e.g. unhealthy diet, physical inactivity, smoking), non-modifiable risk factors (e.g. age, heredity), and intermediate risk factors (e.g. abnormal blood lipids, raised blood pressure) [13], which may additionally contribute to the overall disease risk. Modifiable risk factors are well established for many individual cardiometabolic and chronic pulmonary diseases [14], but their contribution alongside intermediate risk factors to multimorbidity in US adults aged 18–79 years has not been estimated using PAFs. Because cardiometabolic and chronic pulmonary diseases often co-occur and are caused by multiple risk factors, quantifying the contribution of risk factors to overall disease risk using PAFs will be helpful to inform strategies aimed at maximizing the prevention of these diseases. Prior studies suggest greater multimorbidity in marginalized populations, such as homeless individuals, minorities, and adults with lower socioeconomic status and educational attainment [10, 15–18]. Thus, reducing healthcare disparities and targeting cost-effective interventions aimed at reducing common risk factors could have a profound impact on multimorbidity prevention.

The objectives of this study were to determine the prevalence of the multimorbidity of cardiometabolic and chronic pulmonary diseases and to quantify the preventable disease burden through the contribution of simultaneous risk factors on the multimorbidity of these diseases. We analyzed nationally representative data of community-dwelling adults aged 18–79 years, from the National Health and Nutrition Examination Survey (NHANES). Within the broader scope of cardiometabolic and chronic pulmonary diseases, we examined four common disease groups that are associated with high mortality: cardiovascular disease (CVD), diabetes mellitus, chronic kidney disease (CKD), and chronic obstructive pulmonary disease (COPD). 

## Materials and methods

### Study population

Participants included non-pregnant adults aged 18–79 years in the US NHANES 2007–2012, a nationally representative, cross-sectional survey of the resident civilian, non-institutionalized population. The survey design and analytic methods have been previously described [19–21]. Briefly, individuals participated in home interviews along with standardized anthropometric, laboratory, and pulmonary function test assessments in mobile examination centers (MECs). Participants were selected through a complex multistage-probability sampling design, and we used MEC weights in the analysis. Our analytic sample combined data from three survey periods: 2007–2008, 2009–2010, 2011–2012 to yield the final, cross-sectional sample. Overall, unweighted response rates ranged from 70% to 77% across these three survey periods [22]. All participants provided free and informed consent. The Centers for Disease Control and Prevention’s National Center for Health Statistics institutional review board approved the protocol. 

### Chronic disease assessment and measurement

In this analysis, we defined multimorbidity as the co-occurrence of two or more cardiometabolic and chronic pulmonary disease groups, and examined multimorbidity across the following four disease groups: CVD, diabetes mellitus, CKD, and COPD. We used self-reported physician diagnoses and additional self-reported medication use, laboratory, and procedural information as available in NHANES. Disease groups were defined as follows:

- CVD groups included self-reported physician-diagnosed congestive heart failure, coronary heart disease, angina, heart attack, or stroke, as used in the standardized combined questionnaire to define CVD in NHANES.
- Diabetes mellitus was based on self-reported physician-diagnosed diabetes, self-reported use of diabetes medication, such as insulin or metformin, and/or a measured serum hemoglobin A1c (HbA1c) ≥6.5%. HbA1c was measured by high-performance liquid chromatography, which is standardized to the National Glycohemoglobin Standardization Program [23].
- CKD was defined using the National Kidney Foundation/Kidney Disease Outcomes Quality Initiative guidelines [24]. Glomerular filtration rate (GFR) was estimated using the CKD Epidemiology Collaboration equation [25]. Participants were classified as having CKD if they had an estimated GFR <60 mL/min/1.73 m^2^ or an estimated GFR ≥60 mL/min/1.73 m^2^ with albuminuria (albumin-to-creatinine ratio ≥3.4 mg/mmol [30 mg/g]) [26].
- COPD was based on self-reported physician-diagnosed chronic bronchitis or emphysema, or supplemental oxygen use. We additionally included the gold standard diagnosis of COPD with pulmonary function testing based on the Global Initiative for Chronic Obstructive Lung Disease (GOLD) criteria [27]: forced expiratory volume that can be blown out in 1 second (FEV_1_) divided by forced vital capacity (FVC) less than 0.7 (i.e. FEV_1_/FVC <0.7), or FEV_1_/FVC ratio less than the lower limit of normal.

### Assessment and measurement of risk factors

Non-modifiable risk factors included demographic factors (age [28], sex [29], race/ethnicity [30]), and upstream, contextual risk factors for disease (poverty) [31]. Age, sex, race/ethnicity, and poverty were measured by self-report. As a proxy for socioeconomic status, we included poverty, defined using the income-to-poverty ratio, a ratio of family income, and level of poverty [32]. We categorized income-to-poverty ratio into tertiles, in which ratios less than 1.5 (i.e. <150% of the federal poverty threshold) indicated the poorest individuals in the study.

For modifiable risk factors, we included the following intermediate biologic risk factors [13]: overweight/obesity [33], elevated blood pressure [34], and abnormal lipids [35, 36], with their respective measures in this study including body mass index (BMI), hypertension, total cholesterol and high-density lipoprotein (HDL) cholesterol. In addition to these measures that reflect upstream factors, such as diet and physical activity, we included smoking [37] as a modifiable risk factor.

- Overweight/obesity: BMI was defined by clinical measures of weight and height (kg/m^2^) and categorized into underweight (<18.5 kg/m^2^), normal weight (18.5–24.9 kg/m^2^), overweight (25.0–29.9 kg/m^2^), and obese (≥30.0 kg/m^2^) [38]. Underweight estimates were not shown due to small sample sizes (*n*=90 participants who met inclusion criteria) as suggested by NHANES recommendations for reliable estimates [21].
- Hypertension was identified by self-reported physician-diagnosed hypertension and the standard clinical diagnosis of hypertension available through systolic and diastolic blood pressure readings, which were each measured up to four times and averaged. Hypertension was defined as mean systolic blood pressure ≥140 mmHg or mean diastolic blood pressure ≥90 mmHg.
- Dyslipidemia: high total cholesterol and low HDL cholesterol were based on standard definitions of laboratory measures (total cholesterol ≥200 mg/dL or HDL cholesterol <40 mg/dL for men and <50 mg/dL for women).
- Smoking was based on self-report using both the lifetime smoking variable (ever smoked at least 100 cigarettes) in combination with current smoking status and categorized as current, former, or never smoker.

### Statistical analysis

Between 2007 and 2012, 29,353 individuals participated in NHANES. Among this pool, 12,676 participants (43%, unweighted sample) were excluded based on age (<18 years or >79 years) or current pregnancy, with 16,677 participants remaining in the sample. We used a complex sampling design in all analyses. Prevalence estimates of the four disease group outcomes (CVD, diabetes, CKD, and COPD) were stratified by non-modifiable and modifiable risk factors including age, sex, race/ethnicity, poverty, BMI, smoking status, hypertension, total cholesterol, and HDL cholesterol. Chi-square tests were used to compare those who developed disease (e.g. CVD) with those who did not develop disease across categories of risk factors. All significance levels were set at *p* <0.05.

We conducted a multivariate, multiple imputation procedure to impute values for missing disease outcomes. Missing data were greatest for COPD (21.3% of the sample) compared with the other three diseases (CKD 10.6%, CVD 5.3%, and diabetes 0%), similar to existing reports of missing data in NHANES [39]. A weighted sequential hot-deck imputation was performed in SUDAAN [40]. We replaced missing values by taking values from observations with no missing values, with a single donor providing values for the missing outcome of a single recipient. The sample size for the imputed dataset was 16,676, which was used to estimate the prevalence of co-occurring disease combinations in multimorbidity. Results were age-standardized [41] to the 2010 US census population using the age categories 18–39, 40–54, 55–64, and 65–79 years.

Multivariate regression analyses were used to calculate risk ratios (RRs), PAF estimates for modifiable risk factors, and the 95% confidence intervals (CIs) of the PAF using predicted marginals obtained from the RLOGIST (logistic regression) procedure in SUDAAN that adjusted for both modifiable and non-modifiable risk factor covariates. Adjusted RRs were calculated using the dichotomous multimorbidity outcome (two or more conditions versus one or none) and the prevalence of the risk factor among those with multimorbidity. The adjusted PAF (PAF_adj_) was calculated as follows: (number exposed cases/total cases)*[(adjusted RR – 1)/adjusted RR] [36] and using Bonferroni correction for 95% CI estimation [42] (see Supplementary Methods). Multivariate regression excluded participants for missing risk factor data, yielding regression estimates based on 13,331 participants, weighted to represent 180,807,323 US adults. Interactions between risk factors could not be assessed due to the limited sample size. To examine potential bias from missing outcome data, we compared multimorbidity prevalence estimates and PAFs from imputed data, as provided in the results section, with results from non-imputed data (Supplementary Tables 1 and 2), and bias was not detected. All analyses were based on SAS version 9.3 and SUDAAN version 11.0. 

## Results

In a nationally representative sample of non-institutionalized US adults, 17.3% had COPD, 11.6% had CKD, 10.6% had diabetes, and 6.9% had CVD (Table 1). For any disease, the prevalence estimates were significantly greater across older age categories (*p* <0.001). While the prevalence of any of the four disease groups was greatest in adults aged 65–79 years (CVD 23.8%, diabetes 25.9%, CKD 33.9%, COPD 35.3%), younger and middle-aged adults aged 18–39 years also developed chronic diseases with a similar pattern to that of older adults (CVD 1.2%, diabetes 2.6%, CKD 5.9%, COPD 10.4%). Across race/ethnicity categories for the total sample, non-Hispanic blacks had the greatest prevalence of CVD (8.3%), diabetes (16.3%), and CKD (15.0%) compared with non-Hispanic whites, Hispanics, and those who were classified as other race or multiracial (all *p* <0.001). The prevalence of each disease group, except COPD, was significantly greater in individuals with a lower income-to-poverty ratio (*p* <0.0001 across tertiles of income-to-poverty ratios for those with CVD, diabetes, and CKD) (Table 1).

**Figure 1:**
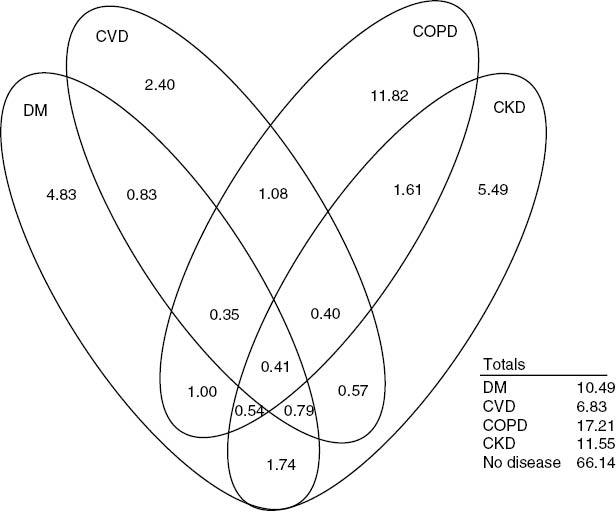
Venn diagram of prevalence estimates across 11 unique combinations of cardiometabolic and chronic pulmonary disease groups among US adults aged 18–79 years in the National Health and Nutrition Examination Survey, age-standardized percent prevalence 2007–2012.Age-standardized to 2010 US census population. Unweighted sample size *N*=16,676. Weighted sample size *N*=214,841,629. CKD, chronic kidney disease; COPD, chronic obstructive pulmonary disease; CVD, cardiovascular disease; DM, diabetes mellitus.

The prevalence estimates of co-occurring disease clusters were assessed (Table 2). The three most common dyads of diseases were (i) diabetes and CKD, (ii) CKD and COPD, and (iii) CVD and COPD. Among adults with CVD, over two-thirds had multimorbidity from co-occurring diabetes, CKD, and/or COPD. Among adults with diabetes, over half had cardiometabolic or chronic respiratory multimorbidity, similar to the proportion of adults with CKD with these co-morbidities. Among adults with COPD, nearly one-third had multimorbidity from co-occurring CVD, diabetes, and/or CKD. The prevalence estimates of specific disease combinations are provided in Figure 1, illustrating the 11 different combinations of multimorbidity in this study. The occurrence of multimorbidity was greater with age, from 1.5% (95% CI 1.2–1.9) for adults aged 18–39 years, to 5.9% (95% CI 5.0–6.9) for those aged 40–54 years, 15.0% (96% CI 13.2–16.9) for those aged 55–64 years and 34.8% (95% CI 32.4–37.2) for those aged 65–79 years.

Table 3 provides the age-standardized prevalence levels of zero, one, and two disease-group combinations for various demographic factors and modifiable risk factors. The age-standardized prevalence of multimorbidity of two or more cardiometabolic and chronic pulmonary disease groups among US adults was 9.3% (95% CI 8.8–9.9) (Table 3), using imputed data (see Supplementary Table 1 for results from original, non-imputed data). The distribution of age-standardized multimorbidity across demographic variables differed when compared with the distribution of demographic factors related to having only one chronic disease. Specifically, age-standardized multimorbidity was greatest among non-Hispanic blacks compared with non-Hispanic whites and other race/ethnicities. However, disparities between non-Hispanic blacks and non-Hispanic whites were not statistically significant for the outcome of one chronic condition. Multimorbidity was greatest among those in the lowest tertile of the income-to-poverty ratio compared with the middle or highest tertiles. Multimorbidity was greater among men compared with women.

For modifiable risk factors, age-standardized multimorbidity was highest in obese adults compared with overweight and normal BMI adults (Table 3). There was no significant difference in the prevalence of multimorbidity between adults with normal BMI versus overweight adults. The prevalence of multimorbidity was also greater in current smokers versus former and never smokers, in those with hypertension versus without hypertension, and in those with low HDL cholesterol versus normal HDL cholesterol. 

Table 4 includes the adjusted RRs, PAF_adj_, and 95% CIs of RRs and PAFs for modifiable risk factors, using imputed data (See Supplementary Table 2 for results from original non-imputed data). Across all measured modifiable risk factors, hypertension was the greatest contributor of multimorbidity (PAF_adj_ 38.8%; 95% CI 29.4–47.4). Other important covariates with high PAF_adj_ for multimorbidity were obesity (19.3%; 95% CI 10.2–28.2), current smoking (10.7%; 95% CI 6.6–15.1), former smoking (8.4%; 95% CI 2.5–14.4), and low HDL cholesterol (13.2%; 95% CI 7.9–18.8). Total cholesterol and being overweight did not have a statistically significant PAF_adj_ for multimorbidity. 

## Discussion

We present prevalence and PAF estimates for the multimorbidity of four highly prevalent cardiometabolic and chronic pulmonary disease groups associated with high mortality in a nationally representative sample of non-institutionalized US adults aged 18–79 years. Nearly 10% of adults had two or more co-occurring conditions, with the most prevalent three disease dyads being (i) diabetes and CKD, (ii) CKD and COPD, and (iii) CVD and COPD. Men, non-Hispanic black adults, and adults with the lowest income-to-poverty ratio had a greater burden of multimorbidity compared with women, non-Hispanic white adults, and adults with higher income-to-poverty ratios, respectively, suggesting persistent healthcare disparities. Among all adults, multimorbidity was most attributable to two *intermediate* biologic risk factors, hypertension and obesity, which are often considered as end conditions in other studies, but are both preventable and treatable, and thus considered in this study as modifiable risk factors for which one may quantify their contribution to disease burden. Reducing hypertension and obesity may effectively reduce the burden of multimorbidity in the USA. Our PAF estimates suggest that eliminating hypertension, while holding other risk factors constant, may translate to a one-third reduction in multimorbidity.

We observed differences in risk factors among adults with multimorbidity versus those with no diseases that were not apparent when comparing adults with one disease versus none, suggesting that multimorbidity may confer a distinctive risk phenotype. The accumulation and/or combination of risk factors may reach a threshold effect after which individuals have increased vulnerability and susceptibility to acquiring more diseases, or alternatively, the resulting progression in multimorbidity may reflect failures in self-management or multispecialty-based management with challenges such as medication adherence and therapeutic competition.

Studies on the prevalence of multimorbidity are highly sensitive to the methods used to define multimorbidity, such as a disease count of two or more coexisting conditions, the inventory of available diseases and conditions, and the population of interest. In our sample of nationally representative US adults aged 18–79 years, disease group prevalences for CVD, diabetes, CKD, and COPD were similar to those reported in prior studies using data from NHANES [43–47]. Our multimorbidity prevalence estimates are further consistent with studies that examined dyads of diseases and comorbidity combinations, such as CVD and CKD, CVD and diabetes, and diabetes and CKD [45, 46], and, similar to other studies, are limited to a few select, but highly prevalent, diseases. Future studies may examine disease combinations and interactions, but will require complex modeling for potential higher-order, multiplicative interactions. 

Few studies have examined modifiable risk factors, including intermediate risk factors [11], or quantified their contribution to multimorbidity using adjusted PAFs in nationally representative US adults aged 18–79 years. Van Baal *et al*. examined the prevalence of the co-occurrence of four of the most highly prevalent chronic diseases (diabetes, myocardial infarction, stroke, and cancer) in the Dutch population [48]. The authors demonstrated that disease co-occurrence occurred more frequently than that expected by the individual diseases alone. The authors postulated that co-occurring diseases share common pathways, such as high blood pressure, cholesterol, smoking, and obesity. The co-occurring diseases increased with age, although the authors did not examine other risk factors [48]. In our study, we reported a similar increasing prevalence of multimorbidity with age and other risk factors using a comparable group of diseases, except that we included CKD and COPD and not cancer. 

One cross-sectional study by Fortin *et al.* [49] examined the association between lifestyle factors and the likelihood of multimorbidity. Multimorbidity was defined as a simple count of three or more of 14 potential chronic conditions, among them CVD, diabetes, and COPD. The odds of multimorbidity were greatest for past or current smoking in men, and for non-normal BMI (either underweight or overweight/obesity) in both sexes. However, the odds of multimorbidity did not differ significantly based on socioeconomic status, education, alcohol use, or low fruit and vegetable consumption. In our study, we demonstrated high PAFs for smoking and obesity, similar to the findings of Fortin *et al*. [49]. However, we additionally reported a greater burden of multimorbidity for non-Hispanic black adults and those with a low income-to-poverty ratio, in contrast to the findings of Fortin *et al.* [49]. The differences in socioeconomic status in the two studies might potentially be explained by the categorization of socioeconomic class. While our study did not examine the association between nutrition and multimorbidity, one study reported that greater consumption of vegetables, fruits, and grains was linked to a lower risk of multimorbidity, based on the evaluation of 11 diseases over 5 years [50]. However, a second study reported no association between low fruit and vegetable consumption and the presence of multimorbidity [49].

We note limitations to our study. First, our sample population focused on community-dwelling adults and did not include nursing home residents and adults 80 years and older (who may have a higher prevalence of cognitive impairment and less reliable self-report), which may underestimate disease prevalence. Second, our prevalence of CVD using physician-diagnosed CVD may be subject to recall bias, under-reporting, and under-diagnosis, as confirmatory catheterization and imaging reports are not collected in NHANES. However, our prevalence estimate is similar to prior estimates in NHANES [44]. Third, for all analyses, we used and presented results utilizing multiple imputation due to some missing data to determine COPD or CKD, although we report original, non-imputed data indicating similar results (See supplementary Tables 1 and 2) suggesting lack of bias from missing data. Finally, our study was cross-sectional and cannot be used to ascertain the temporality of risk factors or causality of these risk factors with multimorbidity. Reverse causation for adults with multimorbidity could modestly inflate the modifiable risk factors measured in this study. Future studies will benefit from longitudinal study designs and a more expansive inventory of risk factors.

Multimorbidity is a common but modifiable phenomenon. Our results suggest that targeting two *intermediate* risk factors, hypertension and obesity, might prevent over half of multimorbidity in US adults aged 18–79 years. Additionally, to reduce the widening of health disparities, public health programs and healthcare policies should target marginalized populations disproportionately burdened by multimorbidity, including older adults, non-Hispanic blacks, and adults with a low income-to-poverty ratio. 

## Figures and Tables

**Table 1:** Prevalence of cardiometabolic and chronic pulmonary disease groups among US adults aged 18–79 years in the National Health and Nutrition Examination Survey, 2007–2012.

Characteristic	Cardiovascular disease (*N*=15,789)		Diabetes (*N*=16,677)		Chronic kidney disease (*N*=15,384)		Chronic obstructive pulmonary disease (*N*=13,544)	
	*n*	% (95% CI)	*n*	% (95% CI)	*n*	% (95% CI)	*n*	% (95% CI)
Total with disease	1,469	6.9 (6.3–7.5)	2,463	10.6 (9.8–11.4)	2,483	11.6 (10.9–12.4)	2,829	17.3 (16.3–18.3)
Age, years								
18–39	85	1.2 (0.9–1.5)	207	2.6 (2.1–3.1)	417	5.9 (5.3–6.6)	670	10.4 (9.4–11.5)
40–54	271	5.0 (4.3–5.8)	605	10.5 (9.2–12.0)	460	8.2 (7.0–9.5)	569	14.3 (12.6–16.2)
55–64	379	11.0 (9.5–12.6)	726	18.1 (15.7–20.8)	516	14.2 (12.1–16.5)	587	25.2 (22.6–27.9)
65–79	734	23.8 (21.6–26.1)	925	25.9 (23.4–28.6)	1,090	33.9 (32.3–35.5)	1,003	35.3 (32.8–38.0)
		*p*<0.0001		*p*<0.0001		*p*<0.0001		*p*<0.0001
Sex								
Male	832	7.9 (7.1–8.7)	1,255	11.0 (10.1–12.0)	1,166	10.2 (9.3–11.3)	1,659	19.7 (18.0–21.5)
Female	636	5.9 (5.3–6.7)	1,208	10.1 (9.2–11.2)	1,317	13.0 (12.1–13.9)	1,169	14.9 (13.6–16.3)
		*p*=0.0001		*p*=0.1069		*p*<0.0001		*p*=0.0003
Race								
White/non-Hispanic	703	7.2 (6.5–8.1)	794	9.1 (8.1–10.2)	995	11.2 (10.4–12.1)	1,520	19.6 (18.4–20.9)
Black/non-Hispanic	384	8.3 (7.4–9.3)	740	16.3 (14.8–17.9)	684	15.0 (13.8–16.3)	599	13.8 (12.6, 15.0)
Hispanic	295	4.5 (3.8–5.4)	740	11.8 (10.6–13.2)	623	11.3 (10.0–12.7)	504	9.8 (8.8–11.0)
Other race/multiracial	86	6.3 (4.6–8.4)	189	12.8 (10.0–16.1)	180	10.4 (8.1–13.2)	205	15.5 (12.3–19.4)
		*p*<0.0001		*p*<0.0001		*p*=0.0002		*p*<0.0001
Income-to-poverty ratio								
0.00 to <1.50	659	8.8 (7.9–9.8)	1,004	12.8 (11.5–14.2)	1,021	14.1 (12.9–15.4)	1,125	17.1 (15.3–19.2)
1.50 to <3.50	400	7.5 (6.6–8.5)	707	11.7 (10.5–13.0)	698	11.9 (10.6–13.3)	772	17.9 (16.2–19.7)
≥3.50	277	5.2 (4.4–6.0)	496	8.4 (7.3–9.6)	528	9.6 (8.6–10.7)	715	17.2 (15.7–18.6)
		*p*<0.0001		*p*<0.0001		*p*<0.0001		*p*=0.7413
Body mass index, kg/m^2^								
<18.5 (underweight)	20	4.7 (2.7–8.0)	4	0.6 (0.2–1.7)	56	14.6 (10.3–20.3)	63	19.9 (14.2–27.1)
18.5 to <25 (normal)	261	4.1 (3.5–4.9)	270	3.6 (3.0–4.4)	532	9.5 (8.5–10.6)	894	19.3 (17.3–21.5)
25 to <30 (overweight)	419	6.0 (5.2–6.9)	627	7.2 (6.5–8.0)	678	9.3 (8.3–10.5)	935	17.9 (16.5–19.5)
≥30 (obese)	723	9.9 (8.9–10.9)	1,500	20.0 (18.3–21.7)	1,151	15.1 (13.8–16.5)	896	14.8 (13.6–15.9)
		*p*<0.0001		*p*<0.0001		*p*<0.0001		*p*=0.0001
Smoking status								
Current smoker	385	8.2 (7.1–9.5)	463	9.6 (8.5–10.8)	496	10.8 (9.6–12.1)	902	26.6 (24.6–28.7)
Former smoker	550	11.6 (10.3–13.1)	793	15.2 (13.5–17.0)	730	15.0 (13.3–16.9)	858	23.6 (21.4–25.9)
Never smoker	529	4.8 (4.2–5.4)	1,193	9.6 (8.7–10.6)	1,173	10.6 (9.8–11.5)	991	11.3 (10.3–12.3)
		*p*<0.0001		*p*<0.0001		*p*<0.0001		*p*<0.0001
Hypertension								
Yes (Stages 1 & 2)	1,159	15.3 (14.1–16.5)	1,790	22.0 (20.3–23.8)	1,692	21.2 (20.0–22.5)	1,382	22.3 (20.5–24.2)
No (normal/pre-hypertension)	310	2.7 (2.3–3.1)	673	4.8 (4.3–5.4)	791	6.8 (6.0–7.6)	1,447	14.7 (13.7–15.9)
		*p*<0.0001		*p*<0.0001		*p*<0.0001		*p*<0.0001
Total cholesterol								
High (≥200 mg/dL)	1,042	9.1 (8.5–9.9)	1,762	13.9 (12.8–15.1)	1,626	13.9 (12.9–15.0)	1,677	19.2 (17.8–20.7)
Normal (<200 mg/dL)	412	4.2 (3.6–5.0)	689	6.7 (6.0–7.4)	834	8.9 (8.1–9.8)	1,105	15.0 (13.8–16.3)
		*p*<0.0001		*p*<0.0001		*p*<0.0001		*p*<0.0001
HDL cholesterol								
Low	554	9.0 (8.0–10.1)	1,043	16.1 (14.6–17.7)	893	14.0 (12.8–15.3)	803	16.1 (14.5–17.8)
High	794	5.9 (5.3–6.5)	1,251	7.9 (7.2–8.7)	1,433	10.6 (9.7–11.5)	1,837	18.0 (16.8–19.2)
		*p*<0.0001		*p*<0.0001		*p*<0.0001		*p*=0.0399

*n* represents unweighted counts; *N* represents unweight sample size. Percent and 95% confidence intervals (CIs) are based on weighted counts. *p* values represent significance of those who developed disease (e.g. cardiovascular disease) compared with those who did not develop disease across categories of risk factors. Missing risk factor data were as follows: 1,132 (9.6%) for income-to-poverty ratio; 471 (4.0%) for body mass index; 608 (5.2%) for current smoking status; 3 (<0.1%) for hypertension; 402 (3.4%) for total cholesterol levels; 1,042 (8.9%) for low high-density lipoprotein (HDL) cholesterol levels.

**Table 2:** Age-standardized prevalence of cardiometabolic and chronic pulmonary disease combinations in the National Health and Nutrition Examination Survey, 2007–2012, without missing outcomes or exposures, *N*=16,676 (weighted sample *N*=214,841,629).

Chronic disease(s)	*n*	%	95% CI
No chronic disease	10,265	66.1	65.2–67.1
One chronic disease	4,297	24.5	23.7–25.4
CVD	472	2.4	2.1–2.7
Diabetes	1,055	4.8	4.4–5.3
CKD	1,035	5.5	5.0–6.1
COPD	1,736	11.8	11.1–12.5
Two chronic diseases	1,492	6.8	6.4–7.3
CVD, diabetes	198	0.8	0.6–1.1
CVD, CKD	135	0.6	0.5–0.7
CVD, COPD	176	1.1	0.9–1.4
Diabetes, CKD	474	1.7	1.5–2.0
Diabetes, COPD	206	1.0	0.9–1.2
CKD, COPD	302	1.6	1.4–1.9
Three chronic diseases	525	2.1	1.8–2.4
CVD, diabetes, CKD	213	0.8	0.6–1.0
CVD, diabetes, COPD	85	0.4	0.3–0.5
CVD, CKD, COPD	93	0.4	0.3–0.6
Diabetes, CKD, COPD	135	0.5	0.4–0.7
Four chronic diseases	97	0.4	0.3–0.6

CI, confidence interval; CKD, chronic kidney disease; COPD, chronic obstructive pulmonary disease; CVD, cardiovascular disease.

**Table 3:** Age-standardized prevalence of common cardiometabolic and chronic pulmonary diseases among US adults aged 18–79 years in the National Health and Nutrition Examination Survey, 2007–2012.

	No disease			One disease			Two or more diseases		
	*n*	%	95% CI	*n*	%	95% CI	*n*	%	95% CI
Total individuals	10,265	66.1	65.2–67.1	4,297	24.5	23.7–25.4	2,114	9.3	8.8–9.9
Sex									
Male	4,995	64.3	62.7–65.9	2,124	25.0	23.7–26.3	1,174	10.7	9.9–11.6
Female	5,270	67.7	66.2–69.1	2,173	24.2	22.9–25.6	940	8.1	7.3–9.0
Race/ethnicity									
White/non-Hispanic	4,173	67.3	66.0–68.5	1,835	24.2	23.1–25.4	921	8.5	7.8–9.3
Black/non-Hispanic	2,144	60.6	58.6–62.5	1,026	26.2	24.4–28.1	585	13.2	12.1–14.4
Hispanic	2,962	66.2	64.3–68.0	1,083	23.8	22.5–25.2	473	10.0	8.9–11.3
Other race/multiracial	987	64.0	59.1–68.5	353	25.6	21.9–29.6	134	10.5	8.0–13.5
Income-to-poverty ratio									
0.00 to <1.50	3,608	59.1	57.7–60.5	1,654	27.1	25.8–28.4	905	13.8	12.7–15.0
1.50 to <3.50	2,738	66.2	64.1–68.3	1,156	24.4	22.6–26.4	603	9.3	8.4–10.3
≥3.50	2,985	69.9	66.9–71.8	1,100	23.1	21.2–25.0	407	7.1	6.4–7.9
Body mass index, kg/m^2^									
18.5 to <25	3,194	68.0	66.3–69.6	1,139	25.4	23.8–27.0	353	6.6	5.7–7.7
25 to <30	3,477	70.0	68.8–71.2	1,296	22.7	21.6–23.9	600	7.3	6.6–8.0
≥30	3,307	61.7	59.9–63.5	1,715	25.4	23.9–26.9	1,072	12.9	11.8–14.1
Smoking status									
Current smoker	2,021	54.9	53.0–56.8	1,082	30.7	28.6–33.0	495	14.4	12.7–16.2
Former smoker	1,766	64.5	62.0–66.9	1,087	25.8	23.5–28.2	762	9.7	8.6–10.9
Never smoker	5,745	71.6	70.3–72.8	1,979	21.1	20.0–22.3	842	7.3	6.6–8.1
Hypertension									
Yes (stages 1 & 2)	2,604	57.4	55.6–59.2	2,103	28.7	27.2–30.3	1,651	13.9	13.0–14.9
No (normal/pre-hypertension)	7,661	71.4	70.1–72.8	2,195	23.1	21.8–24.3	463	5.5	4.8–6.4
Total cholesterol									
High (≥200 mg/dL)	4,935	65.2	63.8–66.5	2,542	24.9	23.6–26.2	1,524	10.0	9.3–10.7
Normal (<200 mg/dL)	5,102	66.5	65.0–68.0	1,691	24.8	23.1–26.5	574	8.7	7.7–9.8
HDL cholesterol									
Low	2,960	61.1	59.1–63.0	1,359	25.6	24.1–27.2	811	13.4	12.4–14.4
Normal	6,649	68.6	67.5–69.6	2,629	23.9	22.9–24.9	1,162	7.6	7.0–8.1

Age-standardized to 2010 US census population. *n* represents unweighted counts. Unweighted sample size *N*=16,676. Weighted sample size *N*=214,841,629. Percentages and 95% confidence intervals (CI) are based on weighted counts. HDL, high-density lipoprotein.

**Table 4:** Population attributable fractions (PAFs) of modifiable risk factors for multimorbidity in US adults aged 18–79 years in the National Health and Nutrition Examination Survey, Age-Standardized, 2007–2012.

Modifiable risk factor	Risk ratio (RR) of multimorbidity		PAF_adj_	
	RR	95% CI	PAF%	95% CI
Body mass index, kg/m^2^				
18.5 to <25	Reference			
25 to <30	0.96	0.80–1.16	−1.05	−6.91–4.33
≥30	1.55	1.29–1.86	19.26	10.15–28.08
Smoking status				
Current smoker	1.75	1.47–2.07	10.68	6.62–15.08
Former smoker	1.29	1.10–1.51	8.40	2.45–14.38
Never smoker	Reference			
Hypertension				
Yes (Stages 1 & 2)	2.10	1.76–2.49	38.84	29.43–47.36
No (normal/pre-hypertension)	Reference			
Total cholesterol				
High (≥200 mg/dL)	1.18	1.02–1.36	11.30	0.13–21.76
Normal (<200 mg/dL)	Reference			
HDL cholesterol				
Low	1.45	1.28–1.65	13.19	7.85-18.81
Normal	Reference			

Analyses were age-standardized to 2010 US census population. Unweighted total sample size *N*=13,331. Weighted total sample size *N*=180,807,323. Estimates were adjusted for risk factors listed within the table, as well as sex, race/ethnicity, and income-to-poverty ratio. CI, confidence interval; HDL, high-density lipoprotein; PAF_adj_, adjusted PAF.
